# Three cases with three different causes of lip and mouth swelling

**DOI:** 10.1186/1939-4551-8-S1-A222

**Published:** 2015-04-08

**Authors:** Amal Assa'ad

**Affiliations:** 1 Cincinnati Children's Hospital Medical Center, USA

## Background

Allergists are often consulted to diagnose and manage patients presenting with edema of the lips, tongue and mouth. It is often feared that the swelling would extend and jeopardize the airway and therefore treatment for presumed anaphylaxis may be initiated. We present three cases where the presentation was thought to due to an allergic reaction, but the etiologies were determined to be different.

## Methods

Three cases with diagnosis of lip, tongue and mouth swelling were reviewed.

## Results

First case is of a previously healthy 8 year old who presented with cough and a fever, and who was initially treated with a macrolide antibiotic develops lip and mouth swelling with blistering lesions leading to sloughing of the buccal mucosa. The presentation was initially thought to be an allergy to the antibiotic, but as the case progressed, a diagnosis of mycoplasma associated buccal mucositis was made. Second case is of a previously healthy 4 year old who presented to the emergency room with pain and swelling of the lips and tongue after drinking from a bottle labeled as containing peach tea. After being treated repeatedly with epinephrine for presumed anaphylaxis to a component of the drink, the case progressed with severe dysphagia and the development of a white coating of the tongue which was diagnosed as the ingestion of a caustic fluid. Third case is an 18 year old with recurrent disfiguring facial swelling. Initially treated with epinephrine for allergic angioedema, the lack of response to epinephrine, the persistence of the swelling over several days led to further work up that made the diagnosis of hereditary angeoneurotic edema despite the absence of a family history.

## Conclusions

Allergist encountering cases of acute onset of edema of the lips, mouth and tongue should entertain a wide differential diagnosis that can include allergic etiologies, idiopathic angioedema or other non allergic disorders as presented above and including complications of mycoplasma infection, ingestion of caustic liquids and hereditary angeoneurotic edema.

